# Anchoring and Sleep Inertia: Sleep Inertia During Nighttime Awakening Does Not Magnify the Anchoring Bias

**DOI:** 10.1027/1618-3169/a000552

**Published:** 2022-10-18

**Authors:** Marie-Lena Frech, Jan Alexander Häusser, Marie-Carolin Siems, David D. Loschelder

**Affiliations:** ^1^ School of Management and Technology, Leuphana University Lüneburg, Germany; ^2^ Justus-Liebig University Gießen, Germany; ^3^ MSH Medical School Hamburg, Germany

**Keywords:** anchoring, sleep inertia, sleepiness, cognitive effort, adjustment

## Abstract

**Abstract.** Many occupational settings require individuals to make important decisions immediately after awakening. Although a plethora of psychological research has separately examined both sleep and anchoring effects on decision-making, little is known about their interaction. In the present study, we seek to shed light on the link between sleep inertia, the performance impairment immediately after awakening, and individuals’ susceptibility to the anchoring bias. We proposed that sleep inertia would moderate participants’ adjustment from anchors because sleep inertia leads to less cognitive effort invested, resulting in a stronger anchoring effect. One hundred four subjects were randomly assigned to an experimental group that answered anchoring tasks immediately after being awakened at nighttime or a control group that answered anchoring tasks at daytime. Our findings replicated the well-established anchoring effect in that higher anchors led participants to higher estimates than lower anchors. We did not find significant effects of sleep inertia. While the sleep inertia group reported greater sleepiness and having invested *less* cognitive effort compared to the control group, no systematic anchoring differences emerged, and cognitive effort did not qualify as a mediator of the anchoring effect. Bayesian analyses provide empirical evidence for these null findings. Implications for the anchoring literature and future research are discussed.


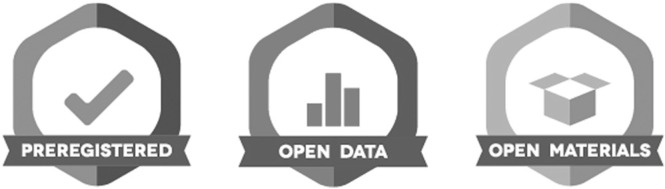


Society is moving more and more toward flexible work schedules, including on-call or standby arrangements, where people only work in the case of an incident and often at unusual work hours (Ferguson et al., 2016). Particularly, people from occupational settings such as the emergency sector, politics, or shift work are frequently required to make decisions soon after awakening. Sleep inertia, or the grogginess and sleepiness felt upon awakening, is associated with detrimental effects on cognitive performance on a variety of tasks, including memory (Achermann et al., 1995), decision-making (Horne & Moseley, 2010), and reasoning (Naitoh et al., 1993).

Despite a multitude of studies on sleep effects, it currently remains unclear whether and how anchoring – a ubiquitous and highly influential phenomenon in human judgment and decision-making – is linked to sleep. The present research seeks to fill this void by investigating how sleep inertia influences individuals’ susceptibility to anchoring.

## Anchoring

Anchoring is defined as the assimilation of judgment to a previously considered standard (i.e., the anchor; Tversky & Kahneman, 1974). The anchoring effect is one of the most robust phenomena in human decision-making as anchors provide orientation for judges’ decisions in various situations of uncertainty. Researchers have proposed a multitude of theories to account for the anchoring effect. One of the most prominent ones is arguably the insufficient adjustment theorizing (Tversky & Kahneman, 1974). Insufficient adjustment argues that individuals use the anchor as a starting point and then adjust away from it until they reach a plausible estimate. Because people terminate the adjustment often prematurely, adjustments are typically *insufficient* (Epley & Gilovich, 2001; see Frech et al., 2020).

Kahneman (2011) described the mental adjustment process away from an anchor as a cognitively demanding process. In line with this theory, Epley and Gilovich (2006) demonstrated that the adjustment-based anchoring effect in estimation tasks is moderated by the ability and willingness to engage in the effortful cognitive adjustment process. Specifically, participants whose cognitive resources were depleted showed less adjustment from the anchor than their nondepleted counterparts, resulting in a stronger anchoring effect (Epley & Gilovich, 2006). In light of these findings, we assume that the quality of people’s estimations and their anchoring susceptibility depend on the effortful cognitive process of adjustment (see Furnham & Boo, 2011). This might be particularly true for self-generated anchors as prior research has argued that effortful adjustment is the underlying mechanism of self-generated anchors but plays a subordinate and less prominent role for experimenter-provided anchors (Epley & Gilovich, 2006; Mussweiler & Strack, 1999).

## Linking Sleep to the Anchoring Bias

Sleep inertia refers to the transition from sleep to wake and is marked by impaired cognitive performance and sleepiness (Trotti, 2017). Sleep inertia occurs regardless of the duration of previous sleep episodes and time of the day (Tassi & Muzet, 2000); however, performance immediately upon awakening is worst during the biological night (Scheer et al., 2008). In addition to sleep inertia, homeostatic and circadian processes shape the relationship between sleep and cognitive functioning (Achermann & Borbély, 2003). Studies that used desynchrony protocols to separate the effects of these processes showed that the impairment caused by sleep inertia can be large, affecting cognitive performance for at least 30 min (Bruck & Pisani, 1999). For example, people experiencing sleep inertia show a much worse performance in tasks requiring visual attention (Burke et al., 2015), reasoning (Naitoh et al., 1993), extraction of information, and tactical planning (Horne & Moseley, 2010). Sleep inertia is associated with severe outcomes in real-world occupational settings, where people are called up to make complex decisions immediately after awakening (e.g., emergency workers, military personnel, airline pilots, politicians, shift workers). For example, Horne and Moseley (2010) demonstrated that junior officers who were awakened due to a simulated sudden crisis in the early morning revealed a markedly impaired decision-making ability compared to participants in a normal awakening *day* control group. Sleep inertia, and in particular the sleepiness that accompanies it, is associated with less effortful decision-making strategies and reduced information processing (Engle-Friedman et al., 2018; Horne & Moseley, 2010). Specifically, previous research showed that sleepiness increases the use of heuristics as they entail less information processing (Dickinson & McElroy, 2019).

These empirical findings notwithstanding, it currently remains unclear whether sleep inertia due to abrupt awakening influences the common and pervasive anchoring heuristic. Based on the assumption that sleep inertia leads to reduced cognitive capacities, one could argue that the anchoring effect is more pronounced because participants adjust *less* from the anchor shortly after awakening during night compared to a daytime control group (Furnham & Boo, 2011). We investigate cognitive effort as a potential underlying mechanism of the anchoring effect, assuming that cognitive resources may be limited due to sleep inertia, and individuals might thus invest less cognitive effort and adjust less away from the anchor after abrupt awakening at night than during the day.We will also explore whether differences between self-generated and experimenter-provided anchors emerge regarding the cognitively demanding adjustment process.

## Contributions and Overview

The aim of the present study is to bridge the psychological literature on the anchoring bias and on sleep-related effects on decision-making by investigating how sleep inertia due to abrupt awakening at night influences (or does not influence) decision-makers’ susceptibility to be anchored. In our pre-registered experiment in the Open Science Framework (OSF; see https://osf.io/w7gtu/), participants were randomly assigned to a control group that answered anchoring tasks at daytime (2:30–4:00 p.m.) or to an experimental group that answered anchoring tasks immediately after being woken up at nighttime (2:30–4:00 a.m.).

## Method

### Pre-Registration

The pre-registration to this study can be found at https://osf.io/w7gtu/; all necessary deviations are detailed in the document “deviations pre-registration” in OSF. We report all measures, manipulations, and exclusions.

### Design and Participants

The experiment followed a 2 sleep inertia (yes vs. no) × 10 anchor level (from low to high) × 12 type of anchor task (estimation anchors, negotiation anchors, self-generated anchors; four tasks each) fully randomized mixed-design, with repeated-measures for the latter factor. Sample size was determined a priori using G*power (Faul et al., 2007). Because, to our knowledge, no prior studies have examined the link between sleep inertia and anchoring, we assumed a conventionally moderate effect size of *f* = 0.25 (*d* = 0.50; Cohen, 1992) for these power analyses. Other parameters were *α* = 0.05, statistical power of 1 − β = .80, and an assumed conservative correlation between the repeated-measures of *r* = 0.6. The sample size analysis led to a minimum sample size of 82 participants (41 per day/nightcondition). Following the comment of an anonymous reviewer, we analyzed the self-generated and experimenter-provided anchors in separate ANOVAs due to the different nature of these anchor tasks. For the repeated-measures analysis of self-generated anchors, a small-to-moderate population effect of *f =* 0.175, *α* = 0.05, and the empirically observed correlation of *r* = 0.1 between measures, the post hoc power for our sample of *N* = 104 was 1 − β = 85.8%. For the repeated-measures analysis of experimenter-provided anchors, a small-to-moderate population effect of *f =* 0.175 could be detected with a power of 1 − β = 95.8% (other parameters: *α* = 0.05, observed *r* = 0.1 between measures, *N* = 104).

Participants were 104 students and faculty members at Leuphana University of Lüneburg, Germany, (*M*_age_ = 23.56, *SD* = 6.89; 73 females). We deliberately oversampled to account for potential outliers – data were not inspected prior to termination of data collection. We excluded outliers whose estimation score exceeded the pre-registered criterion of ±2.5 *SD* from the respective condition mean for each task separately. Thus, participants were only excluded in tasks in which their scores exceeded the criterion, while they remained in the data sample for the other tasks to yield higher power.

### Procedure

Participants were randomly assigned to one of two groups (sleep inertia vs. control group). An e-mail informed participants about the time the experiment would take place; participants were asked to reply to this e-mail with their phone number and to schedule a date. Participants in the experimental group were woken up by a phone call from a member of the author team between 2:30 and 4:00 a.m., while the control group received a phone call between 2:30 and 4:00 p.m. during the day.

Both groups received an e-mail with the link to an online questionnaire which they were asked to complete immediately. This study was conducted via the online survey tool *SoSciSurvey*. A brief introduction to this study was followed by a manipulation check and 12 randomized anchor tasks. Participants also answered questions about their cognitive effort during the tasks, and we exploratory captured a number of possible moderating variables (for a full list of measures and verbatim items, refer to the study’s OSF project). Finally, participants provided demographic information. They were debriefed, thanked, and rewarded with course credit.

### Dependent Variables

#### Manipulation Check

Participants were asked how sleepy they currently felt with a single-item manipulation check (1 = *not at all sleepy*; 7 = *very sleepy*). We used the short version of the Munich Chronotype Questionnaire (Ghotbi et al., 2020) to assess participants’ sleep onset.

#### Anchoring Tasks

We used three different types of anchor tasks that are frequently used in anchoring research – experimenter-provided estimation anchors, experimenter-provided negotiation anchors, and self-generated anchoring tasks (see Epley & Gilovich, 2001; Galinsky & Mussweiler, 2001). There were four tasks per type, 12 tasks in total. For the exact wording, refer to the pre-registration in OSF. To replicate the seminal anchoring effect for experimenter-provided anchors, we used participants’ absolute estimates and counteroffers. To investigate the potential effect of sleep inertia on participants’ anchoring susceptibility for experimenter-provided and self-generated anchors, we used anchor-estimate gaps – that is, the distance between anchors and final estimates (i.e., degree of adjustment away from an anchor; see Simmons et al., 2010).

##### Experimenter-Provided Estimation Anchors

Participants completed four estimation tasks with experimenter-provided anchors. The common two-step anchoring paradigm (Tversky & Kahneman, 1974) first presented the anchor as part of a comparative question (e.g., “Did Mahatma Gandhi die before or after the age of 9?”). Participants were then asked for an absolute estimate (e.g., “How old was Mahatma Gandhi when he died?”; Mussweiler & Strack, 1999). We adapted four items by Jacowitz and Kahneman (1995): *distance between Lisbon and Moscow*, *population of Rome*, *altitude of Mt. Kilimanjaro*, and *number of babies born daily in Germany*.

##### Experimenter-Provided Negotiation Anchors

Four negotiation tasks featured experimenter-provided, first-offer anchors. Participants were introduced to four negotiation scenarios, each with an image of the object of purchase. They were in the buyer role, received a first offer from sellers – the anchor – and were asked to make a counteroffer (Loschelder et al., 2016). Negotiations revolved around a detached house, a necklace, a car, and a salary.

##### Self-Generated Anchors

For the self-generated anchor tasks, participants were asked to provide their own numerical estimate as the starting point, that is, the anchor. They were also asked to indicate why they generated this starting point. Subsequently, they adjusted away from this self-generated anchor and stated their final estimate. We adapted four items by Epley and Gilovich (2001): *freezing point of vodka*, *number of states in the United States in 1840*, *highest recorded body temperature in a human being*, and *gestation period of an elephant*.

##### Manipulation of Anchor Levels

For the experimenter-provided anchors, we used 10 different anchor levels (for all tasks and anchor levels, see pre-registration on OSF). Participants were randomly assigned to one of these levels for each task. The anchor levels varied between tasks to ensure that the extremity of anchors was evenly distributed among participants.

#### Mediator: Cognitive Effort

We measured participants’ self-reported cognitive effort with four self-generated items: “I took a lot of time to find the correct answer,” “I reflected on my answer extensively,” “I roughly estimated my answer” (reversed), “I thought about the correct solution thoroughly” (1 = *completely disagree*; 7 = *completely agree*; Cronbach’s α = .79).

## Results

We analyzed the data with the software R (R Core Team, 2021), IBM SPSS Statistics (Statistical Package for the Social Sciences; version 28.0.0.0), and JASP (Jeffreys's Amazing Statistics Program; version 0.14.1; for the Bayesian analyses).

### Replication of the Anchoring Effect

**Figure 1:**
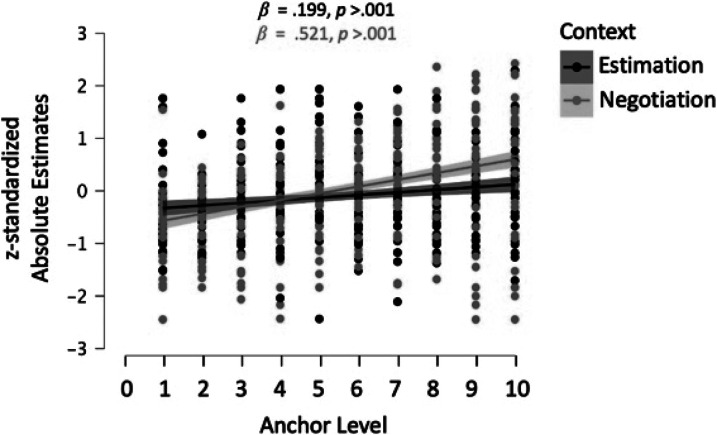
We z-standardized participants' absolute estimates per task and averaged across the four estimation and the four negotiation tasks. The black (pink) line shows an anchoring effect for participants' final estimates across the estimation (negotiation) tasks.

**Figure 2:**
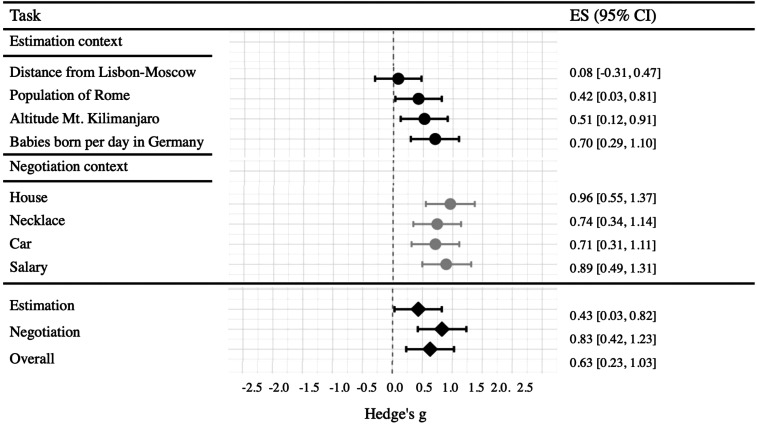
Anchoring effect sizes (Hedge’s g) of the four estimation and the four negotiation tasks. We calculated the 95% confidence interval (95% CI) around the effect size (ES) using the *R* package compute.es. The anchoring effect was, on average, larger for first-offer anchors in negotiations (*g* = 0.83) than for experimenter-provided anchors in estimation tasks (*g* = 0.43).

To investigate the standard anchoring effect, we conducted linear regression analyses for each of the eight tasks with experimenter-provided anchors. We entered anchor level as the predictor and absolute estimates/counteroffers as the dependent variable. As participants generate their own starting points for self-generated anchors, from which they subsequently adjust, we only examined the degree of adjustment as a function of sleep inertia for self-generated anchors (see analyses below). For the estimation anchors, our data revealed significant anchoring effects for all tasks, except for the distance between Lisbon and Moscow, *F*(1, 101) = 0.16, *p* = .690, *R*^2^ = .002, *β* = 0.040. For all other tasks, higher anchors significantly predicted higher estimates, all *p*s < .036, all *R*^2^s > .043, all *β*s > .208 (see Table 1a; Figure 1). For the negotiation anchors, our data revealed significant anchoring effects for all tasks – higher first offers predicted markedly higher counteroffers, all *p*s < .001, all *R*^2^s > .104, all *β*s > .323 (see Table 1b; Figure 1). *R*^2^-values and Bayes factors for the linear regression models (BF_M_) indicated overall larger effects and stronger empirical support for anchoring effects in the negotiation tasks than in the estimation tasks. Overall, a meta-analysis across the eight experimenter-provided tasks corroborated that anchors predicted final values, replicating the seminal anchoring effect (see Figure 2).

**Table 1:** Inferential, Frequentist, and Bayesian Statistics for Anchoring Effects

Task	Anchoring effect				
	*F*	*p*	*R* ^2^	β	BF_M_
(a) Estimation context					
Distance Lisbon–Moscow	0.16	.690	.002	.040	0.22
Population of Rome	4.51	.036	.043	.208	1.52
Altitude of Mt. Kilimanjaro	6.68	.011	.063	.251	3.88
Babies born per day in GER	11.97	.001	.110	.331	35.61
(b) Negotiation context					
House	23.69	<.001	.190	.436	3,794.23
Necklace	13.86	<.001	.121	.347	78.01
Car	12.83	.001	.104	.323	64.69
Salary	20.23	<.001	.168	.410	988.43
*Note*. Linear regression analyses for the estimation and negotiation tasks with experimenter-provided anchors show significant effects for three of the four estimation and for all four negotiation tasks. The reported Bayes factors indicate the extent to which the data support the linear regression model for anchoring (BF_M_).					

### Analyses of Sleep Inertia

#### Manipulation Check

Indicating a successful experimental manipulation of sleep inertia, participants in the experimental group, who were abruptly awakened during night, reported a greater degree of sleepiness (*M* = 5.25, *SD* = 1.17) compared to the control group (*M* = 3.21, *SD* = 1.39), *t*(102) = 8.09, *p* < .001, *d* = 1.59.

#### Sleep Measures

**Figure 3:**
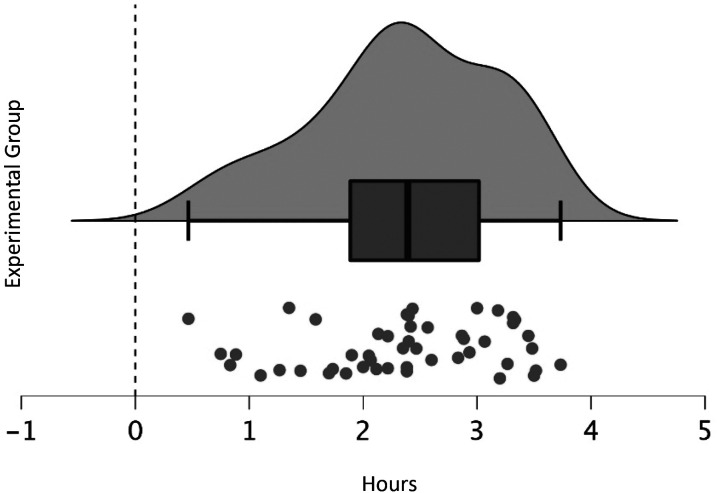
Time between sleep onset and our wake-up call for the experimental group. Using participants’ usual sleep onset (dashed vertical line), we assessed the time elapsed from falling asleep to our wake-up call, indicating that participants were awakened during the first half of the night.

For the experimental group, we assessed the time distance between participants’ usual sleep onset and our wake-up call. On average, we called participants in the sleep inertia group *M* = 2.36 h (*SD* = 0.82) after their regular sleep onset, indicating that they were awakened in the first half of the night when sleep inertia is usually more severe (Dinges & Kribbs, 1991; Figure 3).

#### Does Sleep Inertia Magnify the Anchoring Bias?

We analyzed the data in two different ways to examine whether sleep inertia exerted an effect on participants’ susceptibility to anchoring: First, we conducted two separate repeated-measures ANOVAs for the experimenter-provided anchors and the self-generated anchors. We used participants’ anchor-estimate gaps for these analyses, assuming that people experiencing sleep inertia adjust less from anchors (i.e., smaller adjustment-gaps), which in turn results in a stronger anchoring effect (see Simmons et al., 2010). Second, we conducted separate moderation analyses for experimenter-provided anchors with sleep inertia as a moderator, assuming that higher anchors lead to higher absolute estimates compared to lower anchors and that this difference would be more pronounced when people experience sleep inertia.

##### ANOVA Experimenter-Provided Anchors

**Figure 4:**
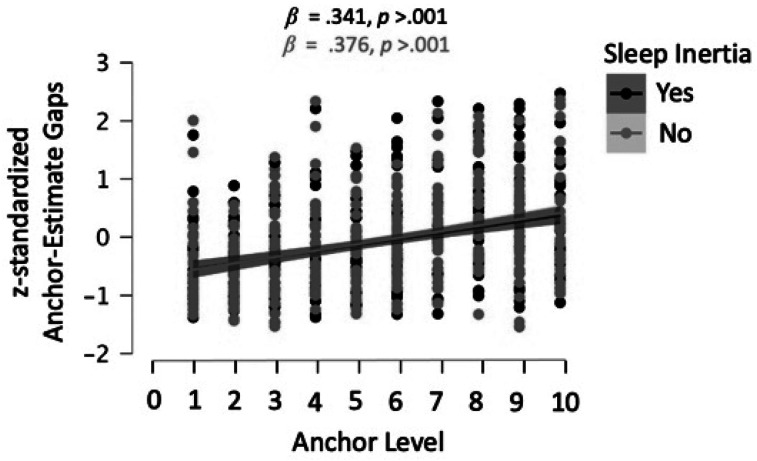
We z-standardized anchor-estimate gaps for each task and averaged across the eight experimenter-provided anchoring tasks. The black line shows anchor-estimate gaps for the experimental group with participants awakened at night. The pink line shows the gaps for the control group. The slopes (see *β* coefficients in black and pink) of these two regression lines did not differ ‐ suggesting no effects of sleep inertia on participants' anchor adjustment.

We conducted a 2 sleep inertia (yes vs. no) × 2 anchor type (estimation vs. negotiation) × 4 tasks (per anchor type) mixed ANOVA with repeated-measures for the two latter within-factors. We used *z*-standardized anchor-estimate gaps as the dependent variable. The results showed no significant main effect of sleep inertia, *F*(1, 88) = 0.02, *p* = .886, η_*p*_^2^ < .01: Participants awakened at night were *not* more susceptible to anchoring than participants in the control group – they did not adjust less away from anchors (see Figure 4). The main effect of anchor type was also not significant, *F*(1, 88) = 0.29, *p* = .594, η_*p*_^2^ < .01; neither were the task main effect, nor any higher-order interaction effect (all *F*s < 1.20, *p*s > .312). Separate Bayesian ANOVAs for each anchor task including sleep inertia and anchor level as independent variables corroborated this pattern of results in that the empirical data strongly supported the null hypothesis of sleep inertia *not* exerting a significant effect (all BF_01_ ranged between 2.97 and 23,830,000; average BF_01_ = 3,227,161.59). The data also supported the null hypothesis for the interaction effect between anchor level and sleep inertia (all BF_01_ ranged between 3.83 and 77.25; average BF_01_ = 35.47; *strong evidence* for the null; Jeffreys, 1961; Lee & Wagenmakers, 2013).

##### ANOVA Self-Generated Anchors

We conducted a 2 sleep inertia (yes vs. no) × 4 tasks mixed ANOVA with repeated-measures for the within-factor tasks. Our results showed no significant main effect of sleep inertia on *z*-standardized anchor-estimate gaps, *F*(1, 94) = 0.70, *p* = .405, η_*p*_^2^ = .01. As with the experimenter-provided anchors, we could not find a significant effect of sleep inertia on anchoring, meaning that participants experiencing sleep inertia did not adjust less from anchors (see Table 2 for descriptives). The main effect of task and the interaction effect were also not significant (*F*s < 0.01, *p*s > .945). Bayesian analyses corroborated this pattern of results in that the data supported the null hypothesis of sleep inertia not exerting an effect (all BF_01_ ranged between 2.39 and 4.81; average BF_01_ = 4.06; *moderate* evidence for the null; Lee & Wagenmakers, 2013).

**Table 2:** Mean (*M*) and Standard Deviation (*SD*) for Anchor-Estimate gaps as a function of Sleep Inertia, Anchor Level, and Anchor Task

Anchor Level	Estimation		Negotiation		Self-generated	
	Sleep Inertia		Sleep Inertia		Sleep Inertia	
	No	Yes	No	Yes	No	Yes
	*M* (*SD*)	*M* (*SD*)	*M* (*SD*)	*M* (*SD*)	*M* (*SD*)	*M* (*SD*)
1	−0.38 (0.96)	−0.40 (0.72)	−0.50 (0.71)	−0.67 (0.60)	−0.03 (0.55)	0.51 (0.64)
2	−0.64 (0.43)	−0.44 (0.62)	−0.52 (0.64)	−0.29 (0.54)		
3	−0.39 (0.68)	−0.42 (0.78)	−0.30 (0.88)	−0.53 (0.51)		
4	−0.08 (0.95)	−0.39 (0.73)	−0.30 (0.59)	−0.22 (0.82)		
5	−0.29 (0.61)	−0.37 (0.58)	−0.24 (0.82)	0.04 (0.70)		
6	−0.08 (0.62)	−0.16 (0.95)	−0.07 (0.64)	0.02 (0.72)		
7	0.11 (0.78)	−0.01 (0.93)	0.20 (0.75)	0.09 (0.86)		
8	0.43 (0.84)	0.60 (0.70)	0.35 (0.90)	0.30 (0.99)		
9	0.35 (0.89)	0.44 (0.96)	0.07 (0.69)	0.37 (1.40)		
10	0.30 (0.78)	0.72 (1.16)	0.75 (1.19)	0.27 (1.17)		
*Note*. *M* and *SD* are used to represent means and *SD*s, respectively. For experimenter-provided anchors, we *z*-standardized and averaged the anchor-estimate gaps for each of the four negotiation and estimation tasks as a function of anchor level and sleep inertia. For self-generated anchors, we *z*-standardized and averaged the four estimation tasks as a function of sleep interia.						

##### Moderation Analysis for Experimenter-Provided Anchors

In a second step, we also investigated the moderating influence of sleep inertia (yes vs. no) on the anchoring effect via moderation analyses using the bootstrapping procedures with 5,000 iterations (process macro; Hayes, 2017; Model 1) for each task. We entered anchor level as the independent variable, sleep inertia (yes vs. no) as the moderator, and absolute estimates as the dependent variable. Self-generated anchors do not allow for this moderation analysis as anchor extremity does not vary. The results revealed no moderation effects across all eight tasks, with a single exception: A significant moderation effect emerged for the estimation task on babies born daily (*b* = 0.087, *SE* = 0.032, CI_95%_ [0.024, 0.151]). However, the anchoring effect was – contrary to our hypothesis – stronger for the control group compared to the awakened group.

### Additional (Mediation) Analyses

We also tested the influence of cognitive effort on participants’ adjustment away from anchors. An exploratory factor analysis using the principle-axis factor extraction method revealed one factor for the scale on cognitive effort with an eigenvalue of 2.49 that explained 62.24% of the total variance and uniformly high factor loadings ranging from λ = .652 to λ = .856. In line with our prediction, the awakened group reported having invested significantly less cognitive effort (*M* = 3.67, *SD* = 1.30) than the control group (*M* = 4.31, *SD* = 1.07), *t*(102) = −2.76, *p* = .007, *d* = 0.54. We then tested whether cognitive effort served as a mediator for the anchoring effect. We ran bootstrapping procedures with 5,000 iterations (process macro; Hayes, 2017; Model 4), entering the experimental condition (sleep inertia vs. control) as an independent variable, cognitive effort as the mediator, and anchor-estimate gaps as the dependent variable. Mediation analyses were conducted separately for each of the 12 tasks. For all 12 tasks, none of the indirect effects were significant; all confidence intervals included zero (all *b*’s < 0.060; all *SE*’s < 0.039) for both self-generated and experimenter-provided anchors. We refrained from testing the moderated mediation model as the mediation through cognitive effort was not significant for any of the tasks and the moderator sleep inertia was also not significant for any of the tasks except the estimation task on the number of babies born daily [and there in the reversed direction]).

## Discussion

The present study addresses a current societal trend toward increasingly flexible work schedules, including on-call or standby arrangements, that requires individuals to make important decisions immediately after awakening (Ferguson et al., 2016). We investigated whether sleep inertia due to abrupt awakening moderates the well-established anchoring effect by comparing anchor susceptibility of participants who made decisions at nighttime after abrupt awaking versus those who made decisions during the day. Moreover, we investigated whether cognitive effort serves as an underlying mechanism of the proposed effect and whether there are differences between self-generated and experimenter-provided anchors.

Our findings replicated the seminal anchoring effect as higher anchors led to higher final estimates compared to lower anchors. Although our sleep inertia manipulation was successful in terms of its intended effect on sleepiness, we did not find empirical support for stronger anchoring under sleep inertia. On the contrary, Bayesian analyses showed moderate to strong empirical evidence for the null hypothesis of no anchor differences as a function of sleep inertia. The results also showed that although, as predicted, the experimental group reported having invested significantly less cognitive effort than the control group, this did not influence or mediate participants’ final estimates.

### Implications and Future Research

There are several reasons that could explain why there was no effect of sleep inertia. Similarly, it is important to discuss factors that we believe did not account for the pattern of results, which should therefore be followed up by future research. First, we wish to argue for the effectiveness of our sleep inertia manipulation: Participants in the group that was awakened during night were indeed markedly sleepier than the control group. Past research has already implemented comparable manipulations that led to significant performance decreases (e.g., Horne & Moseley, 2010).

However, we would like to acknowledge that we did not control for homeostatic and circadian processes that can both influence cognition. While the homeostatic drive or sleep pressure dissipates with time spent asleep, the circadian rhythm promotes sleep at night and alertness during the day (Achermann & Borbély, 2003). Thus, together with sleep inertia, these two processes might have impaired cognitive performance in the experimental group. From this perspective, it can be said that despite the highest possible chance of finding detrimental effects on cognition, we could not find differences in anchoring potency between the experimental group and the control group. Nevertheless, we suggest that future research controls for these different processes, for example, by waking participants in the second half of the night (approximately 4 h after sleep onset), when the homeostatic sleep drive significantly decreases (Daan et al., 1984), and by controlling the circadian rhythm through body temperature (Burke et al., 2015).

We propose that the psychological concept of cognitive effort, which in our data did not function as an underlying mechanism for the anchoring effect, might nonetheless be associated with sleep inertia and sleepiness. Participants tested at night reported having invested significantly less cognitive effort compared to participants tested during the day.

Based on previous anchoring research (e.g., Epley & Gilovich, 2006), which showed that cognitive effort can serve as an underlying mechanism, we had hypothesized that reduced cognitive effort (due to sleep inertia) would result in a stronger anchoring effect, especially for self-generated anchors. It is often argued in the anchoring literature that effortful adjustment is the underlying mechanism for self-generated anchors, while selective accessibility explains the effects for experimenter-provided anchors. For reasons of completeness, there is also research suggesting that cognitive effort can impact anchoring and adjustment independently of the type of anchor (e.g., Chaxel, 2013; Frech et al., 2020; Simmons et al., 2010). In any case, in our experiment, we did not find an influence of sleep inertia on either type of anchor. Importantly, prior studies have shown that both effortful and noneffortful information processing can lead to the assimilation of answers toward anchors (Blankenship et al., 2008). For example, time pressure and attentional load that both reduce cognitive ability to engage in effortful adjustment did not exert an influence on individuals’ anchoring susceptibility (Epley & Gilovich, 2006; Mussweiler & Strack, 1999). The present null finding of sleep inertia thus lends support for the robustness of anchoring across different situational constraints.

Another explanation for the null effect of sleep inertia could be the type of (anchoring) tasks. Previous studies demonstrated that sleepiness mostly impairs decision-making in challenging and complex task environments (Dickinson & McElroy, 2019). Sleep inertia particularly deteriorates decision-making processes involving innovative thinking, spontaneous generation of ideas, and rapid adjustment of behavior (Horne & Moseley, 2010). For example, emergency workers are particularly susceptible to the impairment of sleep inertia as they are required to make important, time-sensitive decisions, high stress, and potentially dangerous tasks shortly after waking (Dawson et al., 2021). In the present study, we used standard anchoring tasks that require general knowledge and participants’ ability to make decisions under uncertainty. While these tasks are well-established and frequently used in anchoring research, they are less complex than the aforementioned scenarios. Thus, our results indicate that differences in cognitive effort caused by sleep inertia did not affect decision-making in less complex scenarios – possibly a true, yet noteworthy null effect (see Friese & Frankenbach, 2020). In all, anchors in rather simple judgment tasks may well be strong during the day *and* while experiencing sleep inertia during nighttime awakening.

### Conclusion

The aim of our research was to investigate whether and how sleep inertia influences participants’ susceptibility to anchoring. Our analyses replicated the seminal anchoring effect but did not find a main or moderation effect of sleep inertia, nor a mediation effect of cognitive effort on anchor adjustments. Hence, the anchoring effect in 12 established tasks was not magnified by the reduced cognitive effort that sleep inertia evoked.

## Notes

**Open Data:** This experiment has been pre-registered at https://osf.io/w7gtu/ (Siems et al., 2022). A full list of data, measures and verbatim items can be found here as well.
